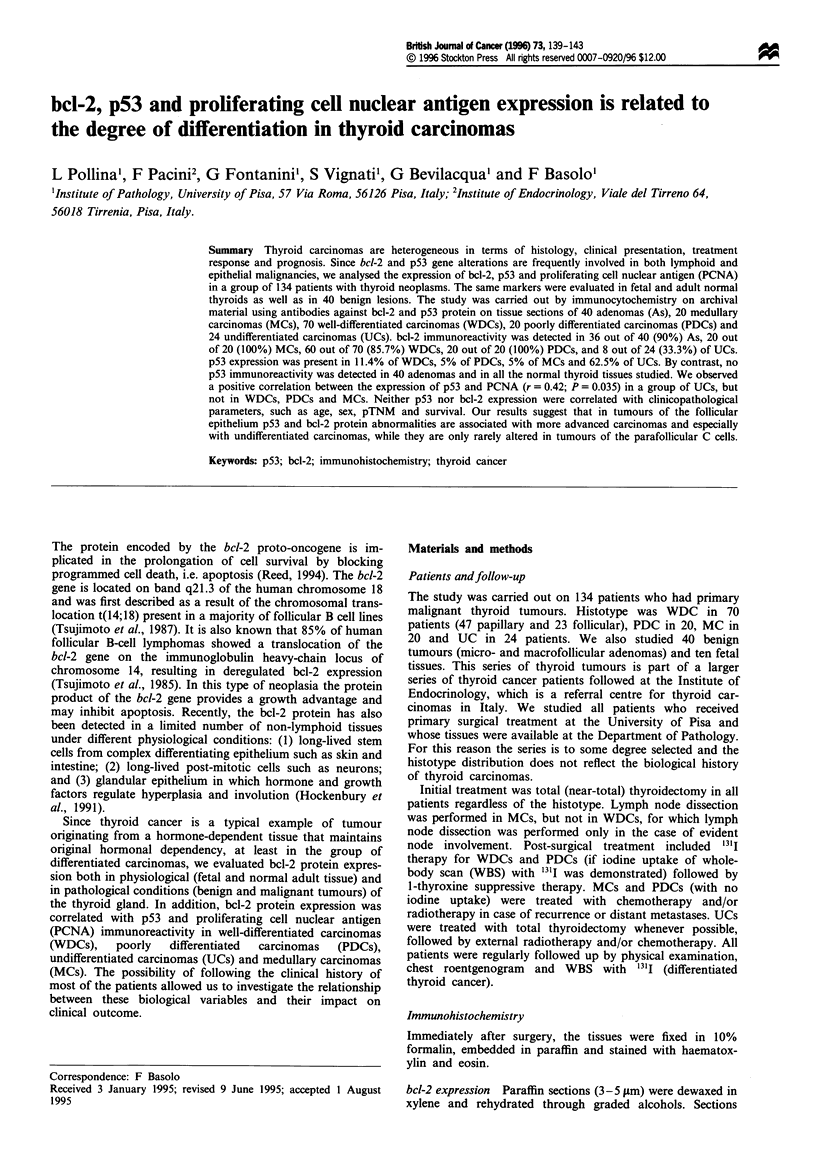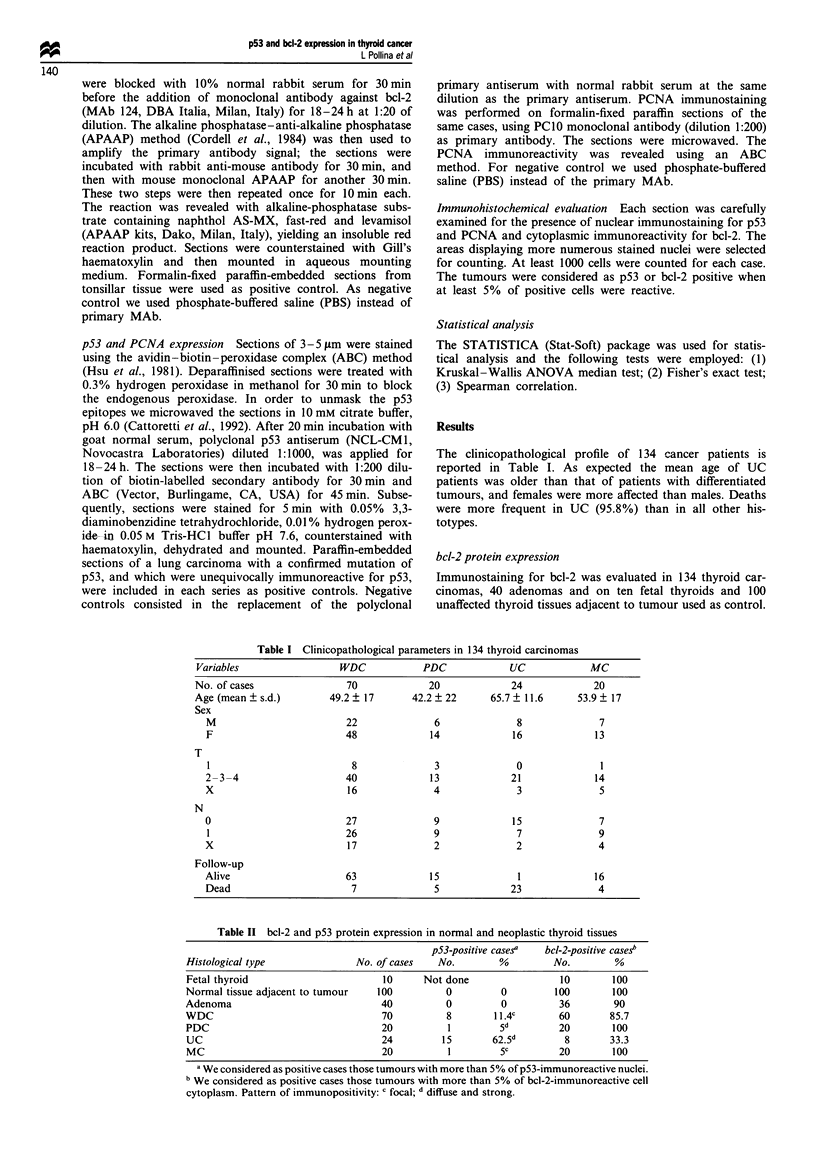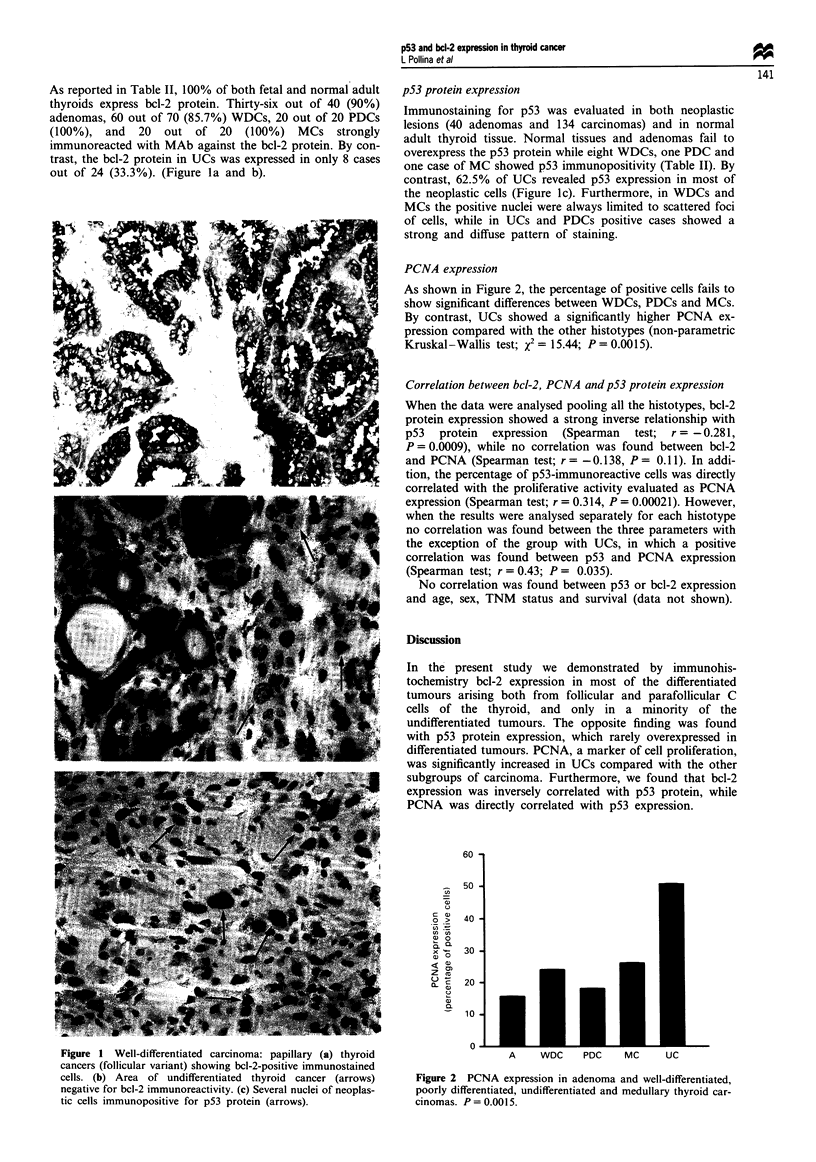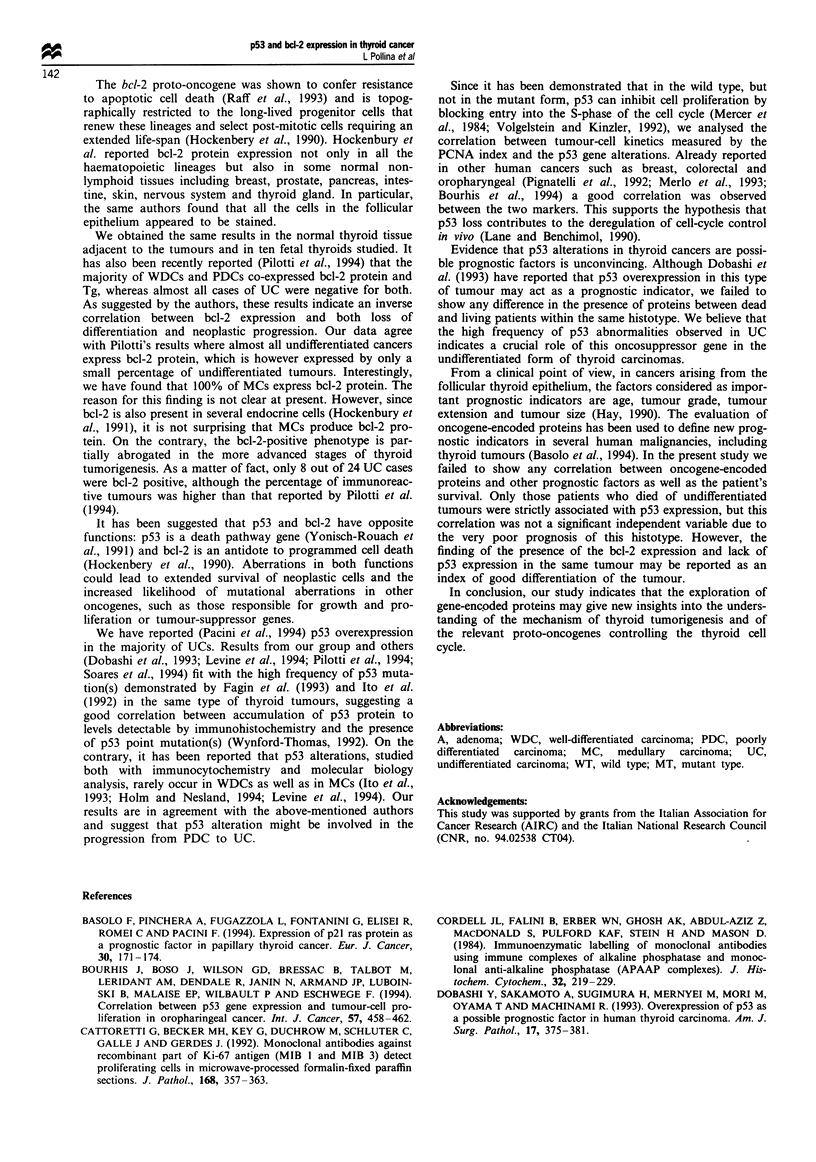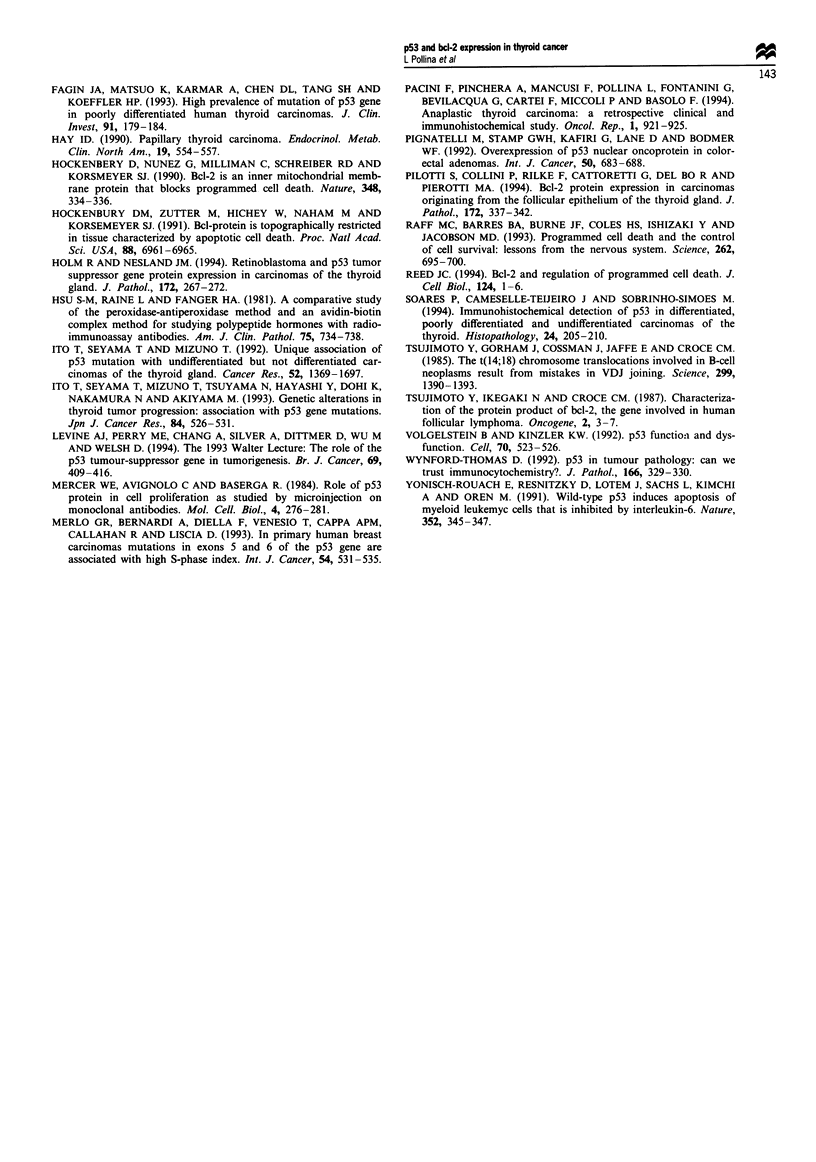# bcl-2, p53 and proliferating cell nuclear antigen expression is related to the degree of differentiation in thyroid carcinomas.

**DOI:** 10.1038/bjc.1996.26

**Published:** 1996-01

**Authors:** L. Pollina, F. Pacini, G. Fontanini, S. Vignati, G. Bevilacqua, F. Basolo

**Affiliations:** Institute of Pathology, University of Pisa, Italy.

## Abstract

**Images:**


British Journal of Cancer (1996) 73, 139–143
© 1996 Stockton Press All rights reserved 0007-0920/96 $12.00

### bcl-2, p53 and proliferating cell nuclear antigen expression is related to the degree of differentiation in thyroid carcinomas

L Pollina[1], F Pacini[2], G Fontanini[1], S Vignati[1], G Bevilacqua[1] and F Basolo[1]

[1]*Institute of Pathology, University of Pisa, 57 Via Roma, 56126 Pisa, Italy;* [2]*Institute of Endocrinology, Viale del Tirreno 64, 56018 Tirrenia, Pisa, Italy.*

**Summary** Thyroid carcinomas are heterogeneous in terms of histology, clinical presentation, treatment response and prognosis. Since *bcl*-2 and p53 gene alterations are frequently involved in both lymphoid and epithelial malignancies, we analysed the expression of bcl-2, p53 and proliferating cell nuclear antigen (PCNA) in a group of 134 patients with thyroid neoplasms. The same markers were evaluated in fetal and adult normal thyroids as well as in 40 benign lesions. The study was carried out by immunocytochemistry on archival material using antibodies against bcl-2 and p53 protein on tissue sections of 40 adenomas (As), 20 medullary carcinomas (MCs), 70 well-differentiated carcinomas (WDCs), 20 poorly differentiated carcinomas (PDCs) and 24 undifferentiated carcinomas (UCs). bcl-2 immunoreactivity was detected in 36 out of 40 (90%) As, 20 out of 20 (100%) MCs, 60 out of 70 (85.7%) WDCs, 20 out of 20 (100%) PDCs, and 8 out of 24 (33.3%) of UCs. p53 expression was present in 11.4% of WDCs, 5% of PDCs, 5% of MCs and 62.5% of UCs. By contrast, no p53 immunoreactivity was detected in 40 adenomas and in all the normal thyroid tissues studied. We observed a positive correlation between the expression of p53 and PCNA ($r = 0.42$; $P = 0.035$) in a group of UCs, but not in WDCs, PDCs and MCs. Neither p53 nor bcl-2 expression were correlated with clinicopathological parameters, such as age, sex, pTNM and survival. Our results suggest that in tumours of the follicular epithelium p53 and bcl-2 protein abnormalities are associated with more advanced carcinomas and especially with undifferentiated carcinomas, while they are only rarely altered in tumours of the parafollicular C cells.

**Keywords:** p53; bcl-2; immunohistochemistry; thyroid cancer

The protein encoded by the *bcl*-2 proto-oncogene is implicated in the prolongation of cell survival by blocking programmed cell death, i.e. apoptosis (Reed, 1994). The *bcl*-2 gene is located on band q21.3 of the human chromosome 18 and was first described as a result of the chromosomal translocation t(14;18) present in a majority of follicular B cell lines (Tsujimoto *et al*., 1987). It is also known that 85% of human follicular B-cell lymphomas showed a translocation of the *bcl*-2 gene on the immunoglobulin heavy-chain locus of chromosome 14, resulting in deregulated bcl-2 expression (Tsujimoto *et al*., 1985). In this type of neoplasia the protein product of the *bcl*-2 gene provides a growth advantage and may inhibit apoptosis. Recently, the bcl-2 protein has also been detected in a limited number of non-lymphoid tissues under different physiological conditions: (1) long-lived stem cells from complex differentiating epithelium such as skin and intestine; (2) long-lived post-mitotic cells such as neurons; and (3) glandular epithelium in which hormone and growth factors regulate hyperplasia and involution (Hockenbury *et al*., 1991).

Since thyroid cancer is a typical example of tumour originating from a hormone-dependent tissue that maintains original hormonal dependency, at least in the group of differentiated carcinomas, we evaluated bcl-2 protein expression both in physiological (fetal and normal adult tissue) and in pathological conditions (benign and malignant tumours) of the thyroid gland. In addition, bcl-2 protein expression was correlated with p53 and proliferating cell nuclear antigen (PCNA) immunoreactivity in well-differentiated carcinomas (WDCs), poorly differentiated carcinomas (PDCs), undifferentiated carcinomas (UCs) and medullary carcinomas (MCs). The possibility of following the clinical history of most of the patients allowed us to investigate the relationship between these biological variables and their impact on clinical outcome.

Correspondence: F Basolo
Received 3 January 1995; revised 9 June 1995; accepted 1 August 1995

### Materials and methods

### *Patients and follow-up*

The study was carried out on 134 patients who had primary malignant thyroid tumours. Histotype was WDC in 70 patients (47 papillary and 23 follicular), PDC in 20, MC in 20 and UC in 24 patients. We also studied 40 benign tumours (micro- and macrofollicular adenomas) and ten fetal tissues. This series of thyroid tumours is part of a larger series of thyroid cancer patients followed at the Institute of Endocrinology, which is a referral centre for thyroid carcinomas in Italy. We studied all patients who received primary surgical treatment at the University of Pisa and whose tissues were available at the Department of Pathology. For this reason the series is to some degree selected and the histotype distribution does not reflect the biological history of thyroid carcinomas.

Initial treatment was total (near-total) thyroidectomy in all patients regardless of the histotype. Lymph node dissection was performed in MCs, but not in WDCs, for which lymph node dissection was performed only in the case of evident node involvement. Post-surgical treatment included $^{131}I$ therapy for WDCs and PDCs (if iodine uptake of whole-body scan (WBS) with $^{131}I$ was demonstrated) followed by l-thyroxine suppressive therapy. MCs and PDCs (with no iodine uptake) were treated with chemotherapy and/or radiotherapy in case of recurrence or distant metastases. UCs were treated with total thyroidectomy whenever possible, followed by external radiotherapy and/or chemotherapy. All patients were regularly followed up by physical examination, chest roentgenogram and WBS with $^{131}I$ (differentiated thyroid cancer).

### *Immunohistochemistry*

Immediately after surgery, the tissues were fixed in 10% formalin, embedded in paraffin and stained with haematoxylin and eosin.

*bcl-2 expression* Paraffin sections (3–5 μm) were dewaxed in xylene and rehydrated through graded alcohols. Sections were blocked with 10% normal rabbit serum for 30 min before the addition of monoclonal antibody against bcl-2 (MAb 124, DBA Italia, Milan, Italy) for 18–24 h at 1:20 of dilution. The alkaline phosphatase–anti-alkaline phosphatase (APAAP) method (Cordell *et al.*, 1984) was then used to amplify the primary antibody signal; the sections were incubated with rabbit anti-mouse antibody for 30 min, and then with mouse monoclonal APAAP for another 30 min. These two steps were then repeated once for 10 min each. The reaction was revealed with alkaline-phosphatase substrate containing naphthol AS-MX, fast-red and levamisol (APAAP kits, Dako, Milan, Italy), yielding an insoluble red reaction product. Sections were counterstained with Gill's haematoxylin and then mounted in aqueous mounting medium. Formalin-fixed paraffin-embedded sections from tonsillar tissue were used as positive control. As negative control we used phosphate-buffered saline (PBS) instead of primary MAb.

*p53 and PCNA expression* Sections of 3–5 μm were stained using the avidin–biotin–peroxidase complex (ABC) method (Hsu *et al.*, 1981). Deparaffinised sections were treated with 0.3% hydrogen peroxidase in methanol for 30 min to block the endogenous peroxidase. In order to unmask the p53 epitopes we microwaved the sections in 10 mM citrate buffer, pH 6.0 (Cattoretti *et al.*, 1992). After 20 min incubation with goat normal serum, polyclonal p53 antiserum (NCL-CM1, Novocastra Laboratories) diluted 1:1000, was applied for 18–24 h. The sections were then incubated with 1:200 dilution of biotin-labelled secondary antibody for 30 min and ABC (Vector, Burlingame, CA, USA) for 45 min. Subsequently, sections were stained for 5 min with 0.05% 3,3-diaminobenzidine tetrahydrochloride, 0.01% hydrogen peroxide in 0.05 M Tris-HCl buffer pH 7.6, counterstained with haematoxylin, dehydrated and mounted. Paraffin-embedded sections of a lung carcinoma with a confirmed mutation of p53, and which were unequivocally immunoreactive for p53, were included in each series as positive controls. Negative controls consisted in the replacement of the polyclonal primary antiserum with normal rabbit serum at the same dilution as the primary antiserum. PCNA immunostaining was performed on formalin-fixed paraffin sections of the same cases, using PC10 monoclonal antibody (dilution 1:200) as primary antibody. The sections were microwaved. The PCNA immunoreactivity was revealed using an ABC method. For negative control we used phosphate-buffered saline (PBS) instead of the primary MAb.

*Immunohistochemical evaluation* Each section was carefully examined for the presence of nuclear immunostaining for p53 and PCNA and cytoplasmic immunoreactivity for bcl-2. The areas displaying more numerous stained nuclei were selected for counting. At least 1000 cells were counted for each case. The tumours were considered as p53 or bcl-2 positive when at least 5% of positive cells were reactive.

### *Statistical analysis*

The STATISTICA (Stat-Soft) package was used for statistical analysis and the following tests were employed: (1) Kruskal–Wallis ANOVA median test; (2) Fisher's exact test; (3) Spearman correlation.

### Results

The clinicopathological profile of 134 cancer patients is reported in Table I. As expected the mean age of UC patients was older than that of patients with differentiated tumours, and females were more affected than males. Deaths were more frequent in UC (95.8%) than in all other histotypes.

### *bcl-2 protein expression*

Immunostaining for bcl-2 was evaluated in 134 thyroid carcinomas, 40 adenomas and on ten fetal thyroids and 100 unaffected thyroid tissues adjacent to tumour used as control.

**Table I** Clinicopathological parameters in 134 thyroid carcinomas

| *Variables* | *WDC* | *PDC* | *UC* | *MC* |
|---|---|---|---|---|
| No. of cases | 70 | 20 | 24 | 20 |
| Age (mean ± s.d.) | 49.2 ± 17 | 42.2 ± 22 | 65.7 ± 11.6 | 53.9 ± 17 |
| Sex | | | | |
| M | 22 | 6 | 8 | 7 |
| F | 48 | 14 | 16 | 13 |
| T | | | | |
| 1 | 8 | 3 | 0 | 1 |
| 2-3-4 | 40 | 13 | 21 | 14 |
| X | 16 | 4 | 3 | 5 |
| N | | | | |
| 0 | 27 | 9 | 15 | 7 |
| 1 | 26 | 9 | 7 | 9 |
| X | 17 | 2 | 2 | 4 |
| Follow-up | | | | |
| Alive | 63 | 15 | 1 | 16 |
| Dead | 7 | 5 | 23 | 4 |

**Table II** bcl-2 and p53 protein expression in normal and neoplastic thyroid tissues

| *Histological type* | *No. of cases* | *p53-positive cases[a]* No. | % | *bcl-2-positive cases[b]* No. | % |
|---|---|---|---|---|---|
| Fetal thyroid | 10 | Not done | | 10 | 100 |
| Normal tissue adjacent to tumour | 100 | 0 | 0 | 100 | 100 |
| Adenoma | 40 | 0 | 0 | 36 | 90 |
| WDC | 70 | 8 | 11.4[c] | 60 | 85.7 |
| PDC | 20 | 1 | 5[d] | 20 | 100 |
| UC | 24 | 15 | 62.5[d] | 8 | 33.3 |
| MC | 20 | 1 | 5[c] | 20 | 100 |

[a] We considered as positive cases those tumours with more than 5% of p53-immunoreactive nuclei. [b] We considered as positive cases those tumours with more than 5% of bcl-2-immunoreactive cell cytoplasm. Pattern of immunopositivity: [c] focal; [d] diffuse and strong.

As reported in Table II, 100% of both fetal and normal adult thyroids express bcl-2 protein. Thirty-six out of 40 (90%) adenomas, 60 out of 70 (85.7%) WDCs, 20 out of 20 PDCs (100%), and 20 out of 20 (100%) MCs strongly immunoreacted with MAb against the bcl-2 protein. By contrast, the bcl-2 protein in UCs was expressed in only 8 cases out of 24 (33.3%). (Figure 1a and b).

**Figure 1** Well-differentiated carcinoma: papillary (**a**) thyroid cancers (follicular variant) showing bcl-2-positive immunostained cells. (**b**) Area of undifferentiated thyroid cancer (arrows) negative for bcl-2 immunoreactivity. (**c**) Several nuclei of neoplastic cells immunopositive for p53 protein (arrows).

*p53 protein expression*

Immunostaining for p53 was evaluated in both neoplastic lesions (40 adenomas and 134 carcinomas) and in normal adult thyroid tissue. Normal tissues and adenomas fail to overexpress the p53 protein while eight WDCs, one PDC and one case of MC showed p53 immunopositivity (Table II). By contrast, 62.5% of UCs revealed p53 expression in most of the neoplastic cells (Figure 1c). Furthermore, in WDCs and MCs the positive nuclei were always limited to scattered foci of cells, while in UCs and PDCs positive cases showed a strong and diffuse pattern of staining.

*PCNA expression*

As shown in Figure 2, the percentage of positive cells fails to show significant differences between WDCs, PDCs and MCs. By contrast, UCs showed a significantly higher PCNA expression compared with the other histotypes (non-parametric Kruskal-Wallis test; $\chi^2 = 15.44$; $P = 0.0015$).

*Correlation between bcl-2, PCNA and p53 protein expression*

When the data were analysed pooling all the histotypes, bcl-2 protein expression showed a strong inverse relationship with p53 protein expression (Spearman test; $r = -0.281$, $P = 0.0009$), while no correlation was found between bcl-2 and PCNA (Spearman test; $r = -0.138$, $P = 0.11$). In addition, the percentage of p53-immunoreactive cells was directly correlated with the proliferative activity evaluated as PCNA expression (Spearman test; $r = 0.314$, $P = 0.00021$). However, when the results were analysed separately for each histotype no correlation was found between the three parameters with the exception of the group with UCs, in which a positive correlation was found between p53 and PCNA expression (Spearman test; $r = 0.43$; $P = 0.035$).

No correlation was found between p53 or bcl-2 expression and age, sex, TNM status and survival (data not shown).

### Discussion

In the present study we demonstrated by immunohistochemistry bcl-2 expression in most of the differentiated tumours arising both from follicular and parafollicular C cells of the thyroid, and only in a minority of the undifferentiated tumours. The opposite finding was found with p53 protein expression, which rarely overexpressed in differentiated tumours. PCNA, a marker of cell proliferation, was significantly increased in UCs compared with the other subgroups of carcinoma. Furthermore, we found that bcl-2 expression was inversely correlated with p53 protein, while PCNA was directly correlated with p53 expression.

**Figure 2** PCNA expression in adenoma and well-differentiated, poorly differentiated, undifferentiated and medullary thyroid carcinomas. $P = 0.0015$.

The *bcl*-2 proto-oncogene was shown to confer resistance to apoptotic cell death (Raff *et al.*, 1993) and is topographically restricted to the long-lived progenitor cells that renew these lineages and select post-mitotic cells requiring an extended life-span (Hockenbery *et al.*, 1990). Hockenbury *et al.* reported bcl-2 protein expression not only in all the haematopoietic lineages but also in some normal non-lymphoid tissues including breast, prostate, pancreas, intestine, skin, nervous system and thyroid gland. In particular, the same authors found that all the cells in the follicular epithelium appeared to be stained.

We obtained the same results in the normal thyroid tissue adjacent to the tumours and in ten fetal thyroids studied. It has also been recently reported (Pilotti *et al.*, 1994) that the majority of WDCs and PDCs co-expressed bcl-2 protein and Tg, whereas almost all cases of UC were negative for both. As suggested by the authors, these results indicate an inverse correlation between bcl-2 expression and both loss of differentiation and neoplastic progression. Our data agree with Pilotti's results where almost all undifferentiated cancers express bcl-2 protein, which is however expressed by only a small percentage of undifferentiated tumours. Interestingly, we have found that 100% of MCs express bcl-2 protein. The reason for this finding is not clear at present. However, since bcl-2 is also present in several endocrine cells (Hockenbury *et al.*, 1991), it is not surprising that MCs produce bcl-2 protein. On the contrary, the bcl-2-positive phenotype is partially abrogated in the more advanced stages of thyroid tumorigenesis. As a matter of fact, only 8 out of 24 UC cases were bcl-2 positive, although the percentage of immunoreactive tumours was higher than that reported by Pilotti *et al.* (1994).

It has been suggested that p53 and bcl-2 have opposite functions: p53 is a death pathway gene (Yonisch-Rouach *et al.*, 1991) and bcl-2 is an antidote to programmed cell death (Hockenbery *et al.*, 1990). Aberrations in both functions could lead to extended survival of neoplastic cells and the increased likelihood of mutational aberrations in other oncogenes, such as those responsible for growth and proliferation or tumour-suppressor genes.

We have reported (Pacini *et al.*, 1994) p53 overexpression in the majority of UCs. Results from our group and others (Dobashi *et al.*, 1993; Levine *et al.*, 1994; Pilotti *et al.*, 1994; Soares *et al.*, 1994) fit with the high frequency of p53 mutation(s) demonstrated by Fagin *et al.* (1993) and Ito *et al.* (1992) in the same type of thyroid tumours, suggesting a good correlation between accumulation of p53 protein to levels detectable by immunohistochemistry and the presence of p53 point mutation(s) (Wynford-Thomas, 1992). On the contrary, it has been reported that p53 alterations, studied both with immunocytochemistry and molecular biology analysis, rarely occur in WDCs as well as in MCs (Ito *et al.*, 1993; Holm and Nesland, 1994; Levine *et al.*, 1994). Our results are in agreement with the above-mentioned authors and suggest that p53 alteration might be involved in the progression from PDC to UC.

Since it has been demonstrated that in the wild type, but not in the mutant form, p53 can inhibit cell proliferation by blocking entry into the S-phase of the cell cycle (Mercer *et al.*, 1984; Volgelstein and Kinzler, 1992), we analysed the correlation between tumour-cell kinetics measured by the PCNA index and the p53 gene alterations. Already reported in other human cancers such as breast, colorectal and oropharyngeal (Pignatelli *et al.*, 1992; Merlo *et al.*, 1993; Bourhis *et al.*, 1994) a good correlation was observed between the two markers. This supports the hypothesis that p53 loss contributes to the deregulation of cell-cycle control *in vivo* (Lane and Benchimol, 1990).

Evidence that p53 alterations in thyroid cancers are possible prognostic factors is unconvincing. Although Dobashi *et al.* (1993) have reported that p53 overexpression in this type of tumour may act as a prognostic indicator, we failed to show any difference in the presence of proteins between dead and living patients within the same histotype. We believe that the high frequency of p53 abnormalities observed in UC indicates a crucial role of this oncosuppressor gene in the undifferentiated form of thyroid carcinomas.

From a clinical point of view, in cancers arising from the follicular thyroid epithelium, the factors considered as important prognostic indicators are age, tumour grade, tumour extension and tumour size (Hay, 1990). The evaluation of oncogene-encoded proteins has been used to define new prognostic indicators in several human malignancies, including thyroid tumours (Basolo *et al.*, 1994). In the present study we failed to show any correlation between oncogene-encoded proteins and other prognostic factors as well as the patient's survival. Only those patients who died of undifferentiated tumours were strictly associated with p53 expression, but this correlation was not a significant independent variable due to the very poor prognosis of this histotype. However, the finding of the presence of the bcl-2 expression and lack of p53 expression in the same tumour may be reported as an index of good differentiation of the tumour.

In conclusion, our study indicates that the exploration of gene-encoded proteins may give new insights into the understanding of the mechanism of thyroid tumorigenesis and of the relevant proto-oncogenes controlling the thyroid cell cycle.

**Abbreviations:**

A, adenoma; WDC, well-differentiated carcinoma; PDC, poorly differentiated carcinoma; MC, medullary carcinoma; UC, undifferentiated carcinoma; WT, wild type; MT, mutant type.

**Acknowledgements:**

This study was supported by grants from the Italian Association for Cancer Research (AIRC) and the Italian National Research Council (CNR, no. 94.02538 CT04).

**References**

BASOLO F, PINCHERA A, FUGAZZOLA L, FONTANINI G, ELISEI R, ROMEI C AND PACINI F. (1994). Expression of p21 ras protein as a prognostic factor in papillary thyroid cancer. *Eur. J. Cancer*, **30**, 171–174.

BOURHIS J, BOSO J, WILSON GD, BRESSAC B, TALBOT M, LERIDANT AM, DENDALE R, JANIN N, ARMAND JP, LUBOINSKI B, MALAISE EP, WILBAULT P AND ESCHWEGE F. (1994). Correlation between p53 gene expression and tumour-cell proliferation in oropharingeal cancer. *Int. J. Cancer*, **57**, 458–462.

CATTORETTI G, BECKER MH, KEY G, DUCHROW M, SCHLUTER C, GALLE J AND GERDES J. (1992). Monoclonal antibodies against recombinant part of Ki-67 antigen (MIB 1 and MIB 3) detect proliferating cells in microwave-processed formalin-fixed paraffin sections. *J. Pathol.*, **168**, 357–363.

CORDELL JL, FALINI B, ERBER WN, GHOSH AK, ABDUL-AZIZ Z, MACDONALD S, PULFORD KAF, STEIN H AND MASON D. (1984). Immunoenzymatic labelling of monoclonal antibodies using immune complexes of alkaline phosphatase and monoclonal anti-alkaline phosphatase (APAAP complexes). *J. Histochem. Cytochem.*, **32**, 219–229.

DOBASHI Y, SAKAMOTO A, SUGIMURA H, MERNYEI M, MORI M, OYAMA T AND MACHINAMI R. (1993). Overexpression of p53 as a possible prognostic factor in human thyroid carcinoma. *Am. J. Surg. Pathol.*, **17**, 375–381.

FAGIN JA, MATSUO K, KARMAR A, CHEN DL, TANG SH AND KOEFFLER HP. (1993). High prevalence of mutation of p53 gene in poorly differentiated human thyroid carcinomas. *J. Clin. Invest*, **91**, 179-184.

HAY ID. (1990). Papillary thyroid carcinoma. *Endocrinol. Metab. Clin. North Am.*, **19**, 554-557.

HOCKENBERY D, NUNEZ G, MILLIMAN C, SCHREIBER RD AND KORSMEYER SJ. (1990). Bcl-2 is an inner mitochondrial membrane protein that blocks programmed cell death. *Nature*, **348**, 334-336.

HOCKENBURY DM, ZUTTER M, HICHEY W, NAHAM M AND KORSEMEYER SJ. (1991). Bcl-protein is topographically restricted in tissue characterized by apoptotic cell death. *Proc. Natl Acad. Sci. USA*, **88**, 6961-6965.

HOLM R AND NESLAND JM. (1994). Retinoblastoma and p53 tumor suppressor gene protein expression in carcinomas of the thyroid gland. *J. Pathol.*, **172**, 267-272.

HSU S-M, RAINE L AND FANGER HA. (1981). A comparative study of the peroxidase-antiperoxidase method and an avidin-biotin complex method for studying polypeptide hormones with radioimmunoassay antibodies. *Am. J. Clin. Pathol.* **75**, 734-738.

ITO T, SEYAMA T AND MIZUNO T. (1992). Unique association of p53 mutation with undifferentiated but not differentiated carcinomas of the thyroid gland. *Cancer Res.*, **52**, 1369-1697.

ITO T, SEYAMA T, MIZUNO T, TSUYAMA N, HAYASHI Y, DOHI K, NAKAMURA N AND AKIYAMA M. (1993). Genetic alterations in thyroid tumor progression: association with p53 gene mutations. *Jpn J. Cancer Res.*, **84**, 526-531.

LEVINE AJ, PERRY ME, CHANG A, SILVER A, DITTMER D, WU M AND WELSH D. (1994). The 1993 Walter Lecture: The role of the p53 tumour-suppressor gene in tumorigenesis. *Br. J. Cancer*, **69**, 409-416.

MERCER WE, AVIGNOLO C AND BASERGA R. (1984). Role of p53 protein in cell proliferation as studied by microinjection on monoclonal antibodies. *Mol. Cell. Biol.*, **4**, 276-281.

MERLO GR, BERNARDI A, DIELLA F, VENESIO T, CAPPA APM, CALLAHAN R AND LISCIA D. (1993). In primary human breast carcinomas mutations in exons 5 and 6 of the p53 gene are associated with high S-phase index. *Int. J. Cancer*, **54**, 531-535.

PACINI F, PINCHERA A, MANCUSI F, POLLINA L, FONTANINI G, BEVILACQUA G, CARTEI F, MICCOLI P AND BASOLO F. (1994). Anaplastic thyroid carcinoma: a retrospective clinical and immunohistochemical study. *Oncol. Rep.*, **1**, 921-925.

PIGNATELLI M, STAMP GWH, KAFIRI G, LANE D AND BODMER WF. (1992). Overexpression of p53 nuclear oncoprotein in colorectal adenomas. *Int. J. Cancer*, **50**, 683-688.

PILOTTI S, COLLINI P, RILKE F, CATTORETTI G, DEL BO R AND PIEROTTI MA. (1994). Bcl-2 protein expression in carcinomas originating from the follicular epithelium of the thyroid gland. *J. Pathol.*, **172**, 337-342.

RAFF MC, BARRES BA, BURNE JF, COLES HS, ISHIZAKI Y AND JACOBSON MD. (1993). Programmed cell death and the control of cell survival: lessons from the nervous system. *Science*, **262**, 695-700.

REED JC. (1994). Bcl-2 and regulation of programmed cell death. *J. Cell Biol.*, **124**, 1-6.

SOARES P, CAMESELLE-TEIJEIRO J AND SOBRINHO-SIMOES M. (1994). Immunohistochemical detection of p53 in differentiated, poorly differentiated and undifferentiated carcinomas of the thyroid. *Histopathology*, **24**, 205-210.

TSUJIMOTO Y, GORHAM J, COSSMAN J, JAFFE E AND CROCE CM. (1985). The t(14;18) chromosome translocations involved in B-cell neoplasms result from mistakes in VDJ joining. *Science*, **299**, 1390-1393.

TSUJIMOTO Y, IKEGAKI N AND CROCE CM. (1987). Characterization of the protein product of bcl-2, the gene involved in human follicular lymphoma. *Oncogene*, **2**, 3-7.

VOLGELSTEIN B AND KINZLER KW. (1992). p53 function and dysfunction. *Cell*, **70**, 523-526.

WYNFORD-THOMAS D. (1992). p53 in tumour pathology: can we trust immunocytochemistry?. *J. Pathol.*, **166**, 329-330.

YONISCH-ROUACH E, RESNITZKY D, LOTEM J, SACHS L, KIMCHI A AND OREN M. (1991). Wild-type p53 induces apoptosis of myeloid leukemyc cells that is inhibited by interleukin-6. *Nature*, **352**, 345-347.